# Nurse Specialising Consultation in Heart Failure: Impact on Drug Titration and Cardiovascular Events in Patients with Heart Failure and Reduced Ejection Fraction

**DOI:** 10.3390/jcm14186681

**Published:** 2025-09-22

**Authors:** Jose Lopez-Aguilera, Jorge Perea-Armijo, Ana Belen Muñoz-Villarreal, Antonia Cepas-Sosa, Luisa Maria Luque-Serrano, Nerea Aguayo-Caño, Gloria Maria Heredia-Campos, Juan Diego Martin-Diaz, Rafael Gonzalez-Manzanares, Juan Carlos Castillo-Dominguez, Manuel Crespin-Crespin, Monica Delgado-Ortega, Martin Ruiz-Ortiz, Dolores Mesa-Rubio, Manuel Pan-Alvarez Osorio, Manuel Anguita-Sanchez

**Affiliations:** 1 Heart Failure Unit, Cardiology Department of Reina Sofia University Hospital, 14004 Cordoba, Spain; 2 Cordoba Biomedical Research Institute, IMIBIC, 14004 Cordoba, Spain

**Keywords:** nurse specialising in heart failure, cardiovascular events, ventricular remodelling, neurohormonal response

## Abstract

**Introduction:** The increasingly active role of nurses in the management of heart failure (HF) has become important in HF units (HCUs). This study aims to determine the effect of opening a specialised HF nursing (NSHF) consultation in a tertiary hospital on drug titration, and its subsequent impact on cardiac remodelling and prognosis. **Methods:** A retrospective cohort study was conducted on patients with HF with reduced ejection fraction (HFrEF) who were treated between 2017 and 2020. Patients who were followed by the NSHF were compared with those who underwent conventional clinical follow-up (non-NSHF), focusing on drug optimisation, echocardiographic parameters, biomarkers, and clinical outcomes in terms of mortality and hospital readmissions for HF. **Results:** A total of 411 patients were analysed, 85 of whom (20.7%) were treated with NSHF. There were hardly any differences in baseline characteristics. At the end of follow-up, the NSHF group had a higher prescription rate of angiotensin receptor–neprilysin inhibitor (+31.7% vs. +23.3%; *p* < 0.001), beta-blockers (+2.4% vs. −5.8%; *p* < 0.001), and sodium glucose co-transporter type 2 inhibitors (+24.7% vs. +17.8%; *p* < 0.001). There was also a higher rate of loop diuretic withdrawal (−16.7% vs. −6.7%; *p* < 0.001). However, no improvement in reverse remodelling or neurohormonal response was observed. Patients treated with NSHF had a lower probability of dying from HF (88.6% vs. 63.3%; *p* = 0.006), but this did not reduce hospital admissions for HF. **Conclusions:** Patients with HFrEF who are cared for through NSHF are more likely to be prescribed drugs that modify the prognosis of the disease. This has an impact on their mortality.

## 1. Introduction

Heart failure (HF) is characterised by its high prevalence and healthcare burden in our country. A large number of patients enter a chronic phase of the disease, which can last for years. This is mainly due to pharmacological developments in recent years that have improved the prognosis and quality of life of patients with this condition [1,2]. Heart failure units (HFUs) have undergone significant development in our country in recent years. Their functions include systematising the diagnosis, the treatment and clinical follow-up of patients with this disease, and coordinating the actions of the different entities and professionals involved in patient care [3].

However, not all HFUs have the same organisational structure or human and material resources [4]. The SEC-Excellent-HF programme in Spain distinguishes three types of HFUs: community, specialised, and advanced. The specialised and advanced HFUs are located within cardiology departments in second- or third-level hospitals. Broadly speaking, they differ in terms of the availability of heart transplant programmes and long-term assist device implantation in advanced centres. All of them must have at least one nurse specialising in HF (NSHF) assigned to the HFUs as part of a multidisciplinary team that includes cardiologists trained in advanced HF, cardiovascular surgeons, anaesthetists, intensivists, and others.

A recently published study [5] specifically analysed the differences between the three types of HFUs defined in Spain by the SEC-Excellent-HF programme. Despite patients in these HFUs having relatively homogeneous baseline characteristics and receiving treatment in accordance with clinical practice guidelines, the study showed that the incidence of serious events per year, including total mortality, hospital admissions, and HF decompensations, was significantly higher in community units than in specialised-advanced units. This could be influenced by the number and training of staff involved in these care programmes for patients with HF.

In HFUs, nursing staff are playing an increasingly active role in managing this disease, mainly due to their contribution to improving patient and family education and care, as well as titrating drugs that modify the prognosis of the disease (angiotensin receptor–neprilysin inhibitors, beta-blockers, sodium glucose co-transporter type 2 inhibitors, and mineralcorticoid receptor antagonists). This study aims to analyse the optimisation and titration of drugs in patients with heart failure and reduced ejection fraction (HFrEF) following the opening of an NSHF in a tertiary hospital. The study will also assess the impact of the clinic’s activities on ventricular remodelling, neurohormonal response, and prognosis in terms of hospital readmissions and mortality in patients with HF.

## 2. Materials and Methods

### 2.1. Study Design and Population

**Figure 1:**
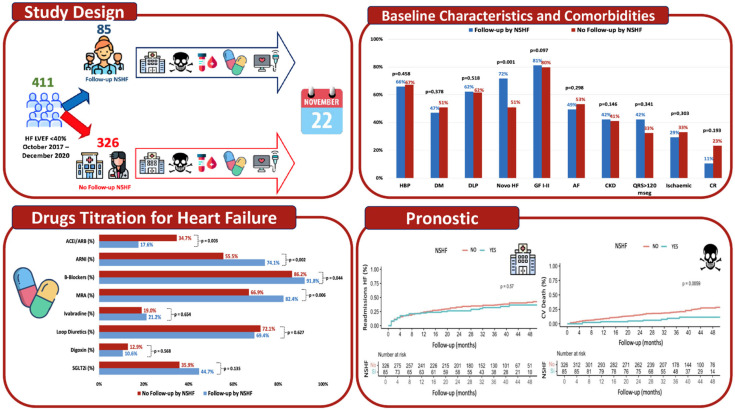
It shows the study design and main results regarding comorbidities, left ventricular remodelling, and prognosis in both cohorts. The results for the cohort followed by a nurse specialising in heart failure are shown in blue, and the results for the cohort without follow-up by a nurse specialising are shown in red. Acronyms: ACEI: angiotensin-converting enzyme inhibitors; AF: atrial fibrillation; ARB: angiotensin receptor blockers; ARNI: angiotensin receptor–neprilysin inhibitors; CKD: chronic kidney disease; CR: cardiac rehabilitation; DLP: dyslipidaemia; DM: diabetes mellitus; HBP: high blood pressure; MRA: mineralocorticoid receptor antagonists; NSHF: nurse specialising in heart failure; SGLT2i: sodium glucose co-transporter type 2 inhibitors.

We present an observational, retrospective, longitudinal, and analytical study of real-world clinical practice. This study included all patients diagnosed with HFrEF who were consecutively treated after hospitalisation or an outpatient consultation for HF symptoms at Reina Sofia Hospital (Cordoba) from October 2017 to December 2020. Follow-up ended in November 2022 due to new admissions for HF or cardiovascular mortality, Figure 1.

A total of 411 patients were included and divided into two follow-up cohorts depending on whether they were treated in the HFU of our hospital and followed up by an NSHF (Cohort 1) or whether they had conventional clinical follow-up without care from a nurse specialising in HF (No NSHF) (Cohort 2). Follow-up visits at NSFH occurred every three weeks, in accordance with our protocol. In the event of clinical deterioration, visits could be at shorter intervals.

The key aspects and differences in the follow-up of HFU patients with respect to conventional clinical follow-up, among the functions of specialised HF nursing, include [3,6]:

- Health education for patients, both at discharge and during follow-up consultations, to promote self-care and shared responsibility in disease management.
- Establishing early follow-up visits with nursing staff and the referring cardiologist shortly after discharge (7–15 days).
- Drug titration with prognostic benefits in HF (angiotensin-converting enzyme inhibitor, angiotensin receptor blockers, angiotensin receptor–neprilysin inhibitor, beta-blockers, diuretics, mineralocorticoid receptor antagonists, sodium glucose co-transporter type 2 inhibitors, and vericiguat) and diuretics, which is based on clinical and analytical criteria.
- Patient telephone access to the HFUs: nursing staff are responsible for answering calls initially and providing a response agreed with the rest of the team.
- Clinical assessment in the event of the patient reporting signs of decompensation, after which the relevant physician is notified for early therapeutic action.
- Day Hospital for HF: nursing staff are responsible for administering intravenous treatments (diuretics, iron, levosimendan…) according to unit protocols and medical prescriptions.

### 2.2. Clinical and Analytical Variables

The aetiology of HF was established according to clinical criteria and the results of additional tests. HF symptoms were defined according to the New York Heart Association functional class. A previous admission for HF was defined as any admission for symptomatic HF requiring intravenous diuretic and/or inotropic treatment between the diagnosis of HFrEF and the start of follow-up. Comorbidities were determined based on data from the initial medical history.

Baseline laboratory parameters were analysed, and previous anaemia was considered if haemoglobin levels were below 13 g/dL in men and below 12 g/dL in women. CKD was defined if the patient met the KDIGO guidelines definition [7].

Data regarding treatment at the beginning and end of the follow-up period were analysed. The use of treatments indicated for HFrEF was recorded, such as renin–angiotensin–aldosterone axis blockers: angiotensin-converting enzyme inhibitors or angiotensin receptor blockers, and mineralocorticoid receptor antagonists; angiotensin receptor–neprilysin inhibitor, beta-blockers, ivabradine, loop diuretics, thiazides, sodium glucose co-transporter type 2 inhibitors, and cardiac resynchronisation therapy (defined as a resynchronisation therapy device implant or physiological pacing in the His bundle or left bundle branch). Implantable cardioverter-defibrillators and percutaneous treatment of mitral regurgitation with an edge-to-edge device were also considered, as well as referral to the cardiac rehabilitation programme. In addition, data on the dosages of pharmacological treatments were collected. Table 1 shows the low, medium, and high doses of the different treatments used in HFrEF.

### 2.3. Echocardiographic Variables

Data from the baseline echocardiogram and the last echocardiogram performed during follow-up were analysed. The following variables of interest were collected: left ventricular end-diastolic volume (mL); left ventricular end-systolic volume (mL); left ventricular end-diastolic diameter (mm); left ventricular end-systolic diameter (mm); and left ventricular ejection fraction (LVEF) (%). The LVEF value was considered valid if it was determined using the Simpson or Teicholtz methods.

### 2.4. Outcome Variables

Prognostic outcomes were assessed based on readmissions for HF and cardiovascular-related mortality.

### 2.5. Statistical Analysis

Quantitative variables were expressed as the mean and standard deviation (if the distribution of values did not conform to normality, then the median and interquartile range were used instead). Categorical variables were expressed as absolute frequencies and percentages. The normality of the quantitative variables was studied using the Shapiro–Wilk test. To compare the two groups, the appropriate parametric or non-parametric tests were used: The chi-squared test or Fisher’s exact test were used for qualitative variables, and the Student’s t-test or the Mann–Whitney U-test were used for quantitative variables. To compare changes in qualitative and quantitative variables within the same group over time, the McNemar and Wilcoxon tests were used, respectively. Event-free survival (readmissions for HF and HF-related mortality) in both groups was analysed using the Kaplan–Meier method, and differences between the survival curves of the two groups were analysed using the log-rank test. All comparisons were two-tailed, and those with *p* < 0.05 were considered statistically significant. Data were collected and analysed using the statistical software packages SPSS v.25 and R 4.2.1.

## 3. Results

### 3.1. Clinical Characteristics and Patient Selection

**Figure 2:**
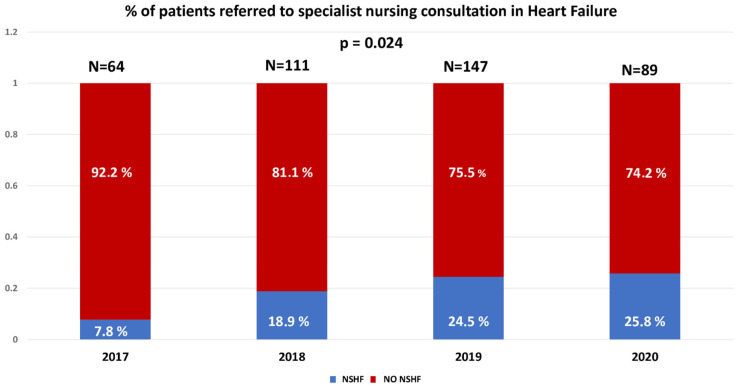
Percentage of patients referred to specialist nursing consultation in heart failure in our centre. Acronyms: NSHF: nurse specialising in HF.

We studied 411 patients diagnosed with HFrEF who were referred to the HFU at our hospital. The patients had a mean age of 67.4 ± 12.6 years, and 74.2% were male. Of these, 85 patients (20.7%) were included in Cohort 1, while 326 patients (79.3%) were included in Cohort 2. As can be seen in Figure 2, the number of patients cared for with NSHF increased progressively throughout the inclusion period from 2017 to 2020. Of the total number of patients treated, 19.5% were referred from outpatient clinics and 80.5% were hospitalised for HF.

Table 2 shows the baseline characteristics of both groups. Cohort 1 had a higher proportion of de novo HF (71.7% vs. 50.0%; *p* < 0.001) and a similar prevalence of atrial fibrillation, chronic renal failure, anaemia, and ischaemic aetiology, but a lower functional class of the disease.

### 3.2. Optimising Treatment for Heart Failure with a Reduced Ejection Fraction and Titration of Prognostic Medications

At the start of the follow-up period, the only difference in prescribing between the two cohorts was that a higher proportion of patients in the NSHF cohort were prescribed angiotensin receptor blockers (83.5% vs. 71.2%; *p* = 0.013). For the other disease-modifying treatments and loop diuretics, there were no statistically significant differences in baseline use (Table 2).

**Figure 3:**
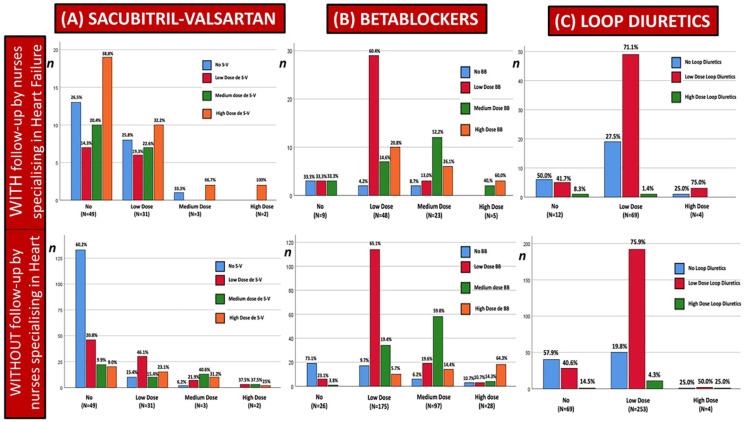
Drug titration in patients with heart failure and reduced ejection fraction, depending on whether or not they have been cared for by a nurse specialising in heart failure. Acronyms: S-V: sacubitril-valsartan; BB: beta-blockers.

By the end of the follow-up period, patients with HFrEF followed by NSHF had achieved better optimisation and titration of disease-specific treatment (Table 3). There was a higher prescription of angiotensin receptor–neprilysin inhibitor (+31.7% vs. +23.3%; *p* = 0.001), β-blockers (+2.4% vs. −5.8%; *p* = 0.001), and sodium glucose co-transporter type 2 inhibitors (+24.7% vs. +17.8%; *p* = 0.001) than in Cohort 2. There was also a greater reduction in the use of loop diuretics (−16.5% vs. −6.7%; *p* = 0.001) and thiazide diuretics (−10.5% vs. +3.3%; *p* = 0.044). There were no differences in the prescription of drugs such as ivabradine, mineralocorticoid receptor antagonists, or digoxin. Figure 3 shows that patients treated by NSHF who are not receiving an angiotensin receptor–neprilysin inhibitor, or who are receiving a low dose initially, undergo a higher level of titration by the end of the follow-up period than those not cared for by NSHF (Figure 3A). A similar trend is observed with β-blockers (Figure 3B), with a clear reduction in the dose of loop diuretics and withdrawal (Figure 3C).

### 3.3. Baseline Echocardiographic Parameters, Cardiac Remodelling, and Neurohormonal Response

In terms of the structural study of the left ventricle, Cohorts 1 and 2 were found to have similar LVEF and ventricular diameters/volumes at the start of the follow-up period (Table 4). In both follow-up groups, LVEF improved, and ventricular diameters/volumes decreased by a similar amount.

Regarding the neurohormonal response analysis, Cohort 1 had comparable baseline NT-proBNP and CA125 levels to Cohort 2. There were no significant differences in neurohormonal response modification at the end of the follow-up period (Table 4).

### 3.4. Prognosis: Readmissions for Heart Failure and Mortality from Heart Failure

**Figure 4:**
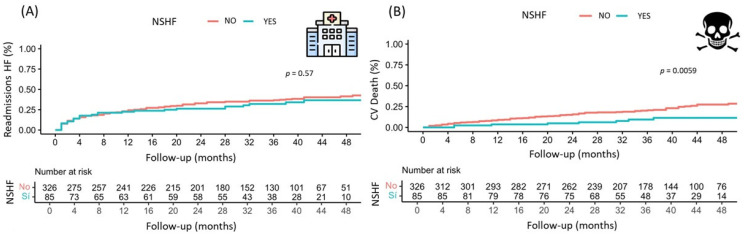
(**A**) Kaplan–Meier curve of time to readmission for HF in patients followed up by a nurse specialising in heart failure and those with conventional follow-up. (**B**) Kaplan–Meier curve of time to cardiovascular mortality in patients followed up by a nurse specialising in heart failure and those with conventional follow-up. Acronyms: CV: cardiovascular; NSHF: nurse specialising in HF.

The median follow-up time for the entire cohort was 36.1 ± 15.9 months. The readmission rate for HF was 37.0% (152 patients), with an HF mortality rate of 21.7% (89 patients) and an all-cause mortality rate of 30.4% (125 patients). There was no statistically significant difference in hospital readmissions for HF between the two comparison groups (see Figure 4), but there was a difference in HF mortality. This was reduced in the group followed by EEIC at the 48-month follow-up (9.4% vs. 11.6%; *p* < 0.001; see Figure 4).

## 4. Discussion

### 4.1. Selection, Classification, and Clinical Characteristics of Patients Followed up in a Specialist Nursing Clinic

Our HFU began operating during the last quarter of 2017. Initially, it consisted of a monographic HF consultation attended by cardiologists. Over the following years, the HFU’s current structure was gradually developed and now includes specialist nursing consultations, a heart failure day hospital, and consultations with cardiologists who specialise in HF. There is also a multidisciplinary team of professionals who care for patients with this condition. The specialist nursing consultation and the day hospital are both staffed by two HF specialist nurses, who have been incorporated gradually over the last few years. The HFU has established referral pathways from the emergency department, the cardiology hospital ward, outpatient consultations, and other specialities. Based on the SEC-Excellent programme’s typology and quality standards for heart failure units [4], the HFU at our centre can be considered an Advanced Specialised Unit. It has access to the latest haemodynamic, electrophysiological, and heart transplantation techniques and therapeutic options, including the implantation of pacing devices and implantable cardioverter-defibrillators. Figure 1 shows that the number of patients treated by NSHF increased progressively during the four years that the study patients were included (2017–2020). However, during this period, the proportion of patients followed by nursing staff remained below 20%. NSHF care was clearly underutilised in the early stages of the HFU at our centre, but this began to change from 2020 onward. Despite the pandemic, referrals to NSHF care increased to over 25%.

Patients referred to the HFU are generally symptomatic and at a high risk of readmission, primarily due to hospital admission for HFrEF. As can be seen in Table 2, the two cohorts have comparable baseline characteristics in terms of clinical features, comorbidities, and cardiac structure. The only variable that could influence the results where there are statistically significant differences is the proportion of de novo HF. There is greater attention to this type of HF in the early stages of the disease by NSHF, probably due to higher referral rates from hospital admissions and greater awareness of the need to refer patients to HFUs for NSHF care. The comorbidity profile is very similar to those published in other recent HFUs studies with similar profiles [5,8,9].

### 4.2. Optimisation and Titration of Treatment for Heart Failure with Reduced Ejection Fraction

In terms of therapeutic characteristics, baseline treatment is also fairly balanced, with no statistically significant differences between the two cohorts in the use of treatments with proven prognostic benefit, such as beta-blockers, renin–angiotensin–aldosterone system blockers, and sodium glucose co-transporter type 2 inhibitors. Compared with the data published by accredited HFUs in the SEC-EXCELENTE programme [5], at our centre, at the end of the follow-up period, there was a higher prescription rate for drugs with prognostic benefits for HF, including angiotensin receptor–neprilysin inhibitor (59% vs. 40.7%), mineralocorticoid receptor antagonists (70.1% vs. 56.9%), beta-blockers (87.3% vs. 80.5%), ivabradine (19.5% vs. 7.9%), and a lower prescription of loop diuretics (71.5% vs. 82.9%). This document does not provide a breakdown of the percentage of thiazide use. However, in our overall series, this type of diuretic is used in less than 20% of cases, a figure that remains unchanged during follow-up. The only treatment that modifies the prognosis of the disease for which the prescription rate in accredited HFUs within the SEC-EXCELENTE programme has not reached is sodium glucose co-transporter type 2 inhibitors (31.1% vs. 44.4%). This is largely due to the dates of the data collection (2017–2020), when the widespread use of sodium glucose co-transporter type 2 inhibitors in patients with HFrEF had not yet been included in clinical practice guidelines. At that time, this pharmacological group was only prescribed to patients with diabetes mellitus. Furthermore, the optimisation of basic HF therapy varies depending on whether the patient is cared for by NSHF staff. Patients cared for by such staff are more likely to be prescribed angiotensin receptor–neprilysin inhibitor, beta-blockers, and sodium glucose co-transporter type 2 inhibitors, and are less likely to be prescribed thiazide and loop diuretics. Regarding two NSHF-led studies, the results in drug prescription rates are inconsistent. The prescription data for beta-blockers in the study by Oyanguren et al. [8] is 89.4% vs. 100%, 93.0% vs. 93.0% for angiotensin-converting enzyme inhibitors/angiotensin receptor blockers/angiotensin receptor–neprilysin inhibitors, and 83.5% vs. 86.8% for mineralocorticoid receptor antagonists. These figures are very similar. However, when our nursing prescription results are compared with those reported by Driscoll et al. [9], it is evident that the prescription of foundational therapy by nurses in this study is significantly lower than in our HFU: beta-blockers (89.4% vs. 77.9%), angiotensin-converting enzyme inhibitors/angiotensin receptor blockers/angiotensin receptor–neprilysin inhibitors (93.0% vs. 56.0%), and mineralocorticoid receptor antagonists (83.5% vs. 38.0%). The results of this study emphasise the importance of drug optimisation in improving patients’ quality of life and prognosis. When optimisation is carried out quickly, as in the STRONG-HF study, it can positively impact patients with HF in the short term without increasing the risk of serious adverse events [10]. This study was conducted at the same time as ours. The prescription of basic therapy and the titration of key drugs in the treatment of this disease are uneven. In our HFU, nurses achieve higher titration levels of angiotensin receptor–neprilysin inhibitor and beta-blockers than patients who are only followed by cardiologists. This is also evident in the ETIFIC study [10], which was a multicentre randomised clinical trial testing the non-inferiority of drug titration by NSHF compared to cardiologists. In this study, nurses achieved higher doses of beta-blockers and angiotensin-converting enzyme inhibitors. This is probably due to better joint follow-up between nurses and cardiologists, where nurses can make multiple visits and patients have greater access to the HFU (both by telephone and in person). This means that signs of decompensation can be identified and treated quickly, either on an outpatient basis or through hospital admission. This early, on-demand access to specialised healthcare professionals is likely to be the real key to the success of these HF patient monitoring programmes [6].

### 4.3. Analysis of Cardiac and Neurohormonal Remodelling

In terms of LVEF improvement, reverse ventricular remodelling and improvement in the neurohormonal response, as measured by NT-proBNP and CA125 levels, there are no differences between patients who are followed by NSHF and those who receive conventional follow-up from a cardiologist. This is most likely because both comparison groups have high rates of disease-modifying drug prescriptions. The same results were obtained in the ETIFIC study [10], which demonstrated that care provided by NSHF is non-inferior to that provided by cardiologists. In this study, significant improvement was observed in both groups in terms of the LVEF, NT-proBNP, New York Heart Association classification, 6-minute walk test, and quality of life at the 6-month follow-up.

### 4.4. The Prognostic Impact of Specialised Nursing Care in Heart Failure

In terms of the outcome results, our findings regarding the reduction in cardiovascular mortality achieved through HF care programmes led by nursing staff are consistent with those of other similar studies [11,12,13]. This demonstrates the impact of nursing care on heart failure, as has been observed in other areas such as smoking cessation, glycaemic control in diabetics, and obesity reduction in patients after acute coronary syndrome, with the associated benefits [14,15,16].

However, the impact on the reduction of hospital admissions for HF has not been demonstrated [11,17,18], most likely due to different causes: First, there is a low rate of readmissions for heart failure in both cohorts. Second, treatment is highly optimised in both groups, demonstrating the high level of specific HF training of the participating cardiologists. Third, the cohort followed by nurses has a higher dose reduction and withdrawal of diuretics (both loop and thiazide), which may lead to an increase in short-term admissions in the group followed by NSHF.

### 4.5. Limitations

The study is single-centre and retrospective, so it has limitations inherent to this type of study. This is a study on the start of the specialist heart failure nursing practice in a tertiary hospital, with an increasing number of patients being referred in recent years, which implies greater experience in its management. In addition, no information is provided on the frequency of patient monitoring (face-to-face or remote) by nurses. On the other hand, there is a low prescription of sodium glucose co-transporter type 2 inhibitors, given that their use as a first line in patients with ICFEr appeared in the 2021 clinical practice guidelines, this period being selected because it was the start of our specialised consultation.

## 5. Conclusions

Patients with heart failure and reduced ejection fraction who are cared for by specialist nurses in our heart failure unit are more likely to receive the drug prescription and titration for the foundational therapy of the disease (renin–angiotensin system and neprilysin inhibitors), as well as greater use of beta-blockers and sodium glucose co-transporter type 2 inhibitors. There is also a greater reduction in thiazide and loop diuretics. These measures have an impact on cardiovascular mortality without improving the LVEF or the neurohormonal response.

## Figures and Tables

**Table 1:** **Doses of the different pharmacological groups used in heart failure with reduced ejection fraction.** Acronyms: ARNI: angiotensin receptor–neprilysin inhibitors; MRA: mineralocorticoid receptor antagonists; QD: once daily; BID: twice daily.

Pharmacological Group	Low Dose	Medium Dose	High Dose
**Loop diuretics**	Furosemide ≤ 120 mg QD		Furosemide > 120 mg QD
**Thiazide diuretics**	Hydrochlorotiazide ≤ 25 mg QD Chlortalidone ≤ 25 mg QD		Hydrochlorotiazide > 25 mg QD Chlortalidone > 25 mg QD
**Betablockers**	Bisoprolol ≤ 2.5 mg QD Carvedilol < 12.5 mg BID Nebivolol ≤ 2.5 mg QD Atenolol < 50 mg QD	Bisoprolol 2.5–5 mg QD Carvedilol 12.5–25 mg BID Nebivolol 2.5–5 mg QD Atenolol 50–75 mg QD	Bisoprolol > 5 mg QD Carvedilol ≥ 25 mg BID Nebivolol > 5 mg QD Atenolol > 75 mg QD
**MRA**	Eplerenone ≤ 25 mg QD Spironolactone ≤ 25 mg QD		Eplerenone > 25 mg QD Spironolactone > 25 mg QD
**Ivabradine**	Ivabradine ≤ 5 mg QD		Ivabradine > 5 mg/QD
**ARNI**	Sacubitril-Valsartan ≤ 24/26 mg BID	Sacubitril-Valsartan 49/51 mg BID	Sacubitril-Valsartan 97/103 mg BID

**Table 2:** **Baseline characteristics.** Acronyms: ACEI: angiotensin-converting enzyme inhibitor; ARB: angiotensin receptor blockers; CKD: chronic kidney disease; Cr: creatinine; CV: cardiovascular; DLP: dyslipidaemia; DM: diabetes mellitus; Hb: haemoglobin; HBP: high blood pressure; HF: heart failure; LVEF: left ventricular ejection fraction; LVEDD: left Ventricular End Diastolic Diameter; LVESD: Left Ventricular End Systolic Diameter; LVEDV: Left ventricular end diastolic volume; LVESV: left ventricular end systolic volume; NSHF: nurse specialising in HF; MRA: mineralocorticoid receptor antagonists.

	HFrEF (*n* = 411)	NSHF YES (*n* = 85)	NSHF NO (*n* = 326)	*p*
**Male sex (%)**	305 (74.2%)	62 (72.9%)	243 (74.5%)	0.431
**Age (years)**	67.4 ± 12.6	66.9 ± 13.8	67.6 ± 12.3	0.677
**CV risk factor** DM HBP DLP Smoking Ex-smoking	202 (49.1%) 275 (66.9%) 254 (61.8%) 50 (12.2%) 163 (39.6%)	40 (47.0%) 56 (65.9%) 53 (62.3%) 8 (9.4%) 33 (38.8%)	162 (50.9%) 219 (67.2%) 201 (61.6%) 42 (12.9%) 130 (39.9%)	0.378 0.458 0.518 0.540 0.530
**DM**	202 (49.1%)	40 (47.0%)	162 (50.9%)	0.378
**HBP**	275 (66.9%)	56 (65.9%)	219 (67.2%)	0.458
**DLP**	254 (61.8%)	53 (62.3%)	201 (61.6%)	0.518
**Smoking**	50 (12.2%)	8 (9.4%)	42 (12.9%)	0.540
**Ex-smoking**	163 (39.6%)	33 (38.8%)	130 (39.9%)	0.530
**HF de novo**	227 (55.2%)	61 (71.7%)	166 (50.9%)	0.001
**Heart failure evolution time (months)**	36.3 ± 66.7	27.6 ± 60.1	38.6 ± 68.3	0.177
**Diagnosis** Hospital admission Outpatient consultation	331 (80.5%) 80 (19.5%)	74 (87.0%) 11 (13.0%)	257 (78.8%) 69 (21.1%)	0.001
**Functional Class** NYHA I NYHA II NYHA III-IV	63 (15.3%) 266 (64.8%) 82 (19.9%)	6 (7.0%) 63 (74.2%) 16 (18.8%)	57 (17.5%) 203 (62.3%) 66 (20.2%)	0.097
**Aetiology** Ischaemic	133 (32.3%)	25 (29.4%)	108 (33.1%)	0.303
**Atrial Fibrillation**	216 (52.5%)	42 (49.4%)	174 (53.3%)	0.298
**QRS (ms)** **QRS > 120 ms** RBBB LBBB	114.6 ± 28.3 142 (34.5%) 35 (8.5%) 107 (26.0%)	122.2 ± 30.7 36 (42.3%) 10 (11.7%) 26 (30.6%)	111.7 ± 26.9 106 (32.5%) 25 (7.7%) 81 (24.8%)	0.031 0.341 0.626
**LVEF (%)** **LVEDD (mm)** **LVESD (mm)** **LVEDV (mL)** **LVESV (mL)**	30.1 ± 6.0 62.6 ± 8.3 52.7 ± 9.1 163.8 ± 60.4 112.1 ± 44.5	30.0 ± 5.8 62.4 ± 7.9 52.8 ± 8.9 170.1 ± 59.2 115.2 ± 42.8	30.1 ± 6.1 62.6 ± 8.5 52.7 ± 9.2 161.7 ± 60.8 111.1 ± 45.1	0.952 0.896 0.952 0.342 0.544
**CKD** **Cr (mg/dL)** **GFR (mL/min)**	173 (42.1%) 1.3 ± 0.7 66.2 ± 26.7	36 (42.3%) 1.2 ± 0.4 68.0 ± 24.5	134 (41.1%) 1.3 ± 0.8 65.8 ± 27.1	0.146 0.196 0.486
**Hb (g/dL)**	13.6 ± 2.1	13.6 ± 2.1	13.6 ± 2.3	0.997
**CA-125 (U/mL)** **NT-proBNP (pg/mL)**	61.8 ± 105.8 10,524.2 ± 23,051.1	31.4 ± 48.8 9596.5 ± 13,056.4	63.4 ± 111.2 7785.2 ± 8501.9	0.070 0.680
**Treatment:** ACEI/ARB ARNI βB Loop diuretics MRA Ivabradine Digoxin SGLT2i Cardiac rehabilitation	235 (57.2%) 141 (34.3%) 376 (91.5%) 330 (80.3%) 303 (73.7%) 88 (21.4%) 49 (11.9%) 76 (18.5%) 32 (7.8%)	43 (50.6%) 36 (42.3%) 76 (89.4%) 73 (85.9%) 71 (83.5%) 16 (18.8%) 8 (9.4%) 17 (20.0%) 9 (10.6%)	192 (58.9%) 105 (32.2%) 300 (92.0%) 257 (78.8%) 232 (71.2%) 72 (22.1%) 41 (12.6%) 59 (18.1%) 76 (23.3%)	0.105 0.053 0.283 0.094 0.013 0.312 0.276 0.396 0.193

**Table 3:** **Modification of drug prescriptions for the treatment of HFrEF in patients cared for by nurses specialising in heart failure vs. patients receiving conventional follow-up**. Acronyms: ACEI: angiotensin-converting enzyme inhibitor; ARB: angiotensin receptor blockers; ARNI: angiotensin receptor–neprilysin inhibitors; NSHF: nurse specialising in heart failure; MRA: mineralocorticoid receptor antagonists; SGLT2i: sodium glucose co-transporter type 2 inhibitors.

	Total of Patients (*n* = 411)				NSHF (Yes) (*n* = 85)				NSHF (No) (*n* = 326)			
	Basal	Final	Difference (%)	*p*	Basal	Final	Difference (%)	*p*	Basal	Final	Difference (%)	*p*
**ACEI/ARB (%)**	235 (57.2%)	128 (31.1%)	−26.1%	0.001	43 (50.6%)	15 (17.6%)	−33.0%	0.001	192 (58.9%)	113 (34.7%)	−24.2%	0.001
**ARNI (%)**	141 (34.3%)	244 (59.4%)	+25.1%	0.001	36 (42.4%)	63 (74.1%)	+31.7%	0.001	105 (32.2%)	181 (55.5%)	+23.3%	0.001
**Beta-blocker (%)**	376 (91.5%)	359 (87.3%)	−4.2%	0.014	76 (89.4%)	78 (91.8%)	+2.4%	0.754	300 (92.0%)	281 (86.2%)	−5.8%	0.001
**MRA (%)**	303 (73.7%)	288 (70.1%)	−3.6%	0.133	71 (83.5%)	70 (82.4%)	−1.1%	1.000	232 (71.2%)	218 (66.9%)	−4.3%	0.125
**Ivabradine (%)**	88 (21.4%)	80 (19.5%)	−1.9%	0.312	16 (18.8%)	18 (21.2%)	+2.4%	0.687	72 (22.1%)	62 (19.0%)	−3.1%	0.164
**Loop diuretics (%)**	330 (80.3%)	294 (71.5%)	−8.8%	0.001	73 (85.9%)	59 (69.4%)	−16.5%	0.009	257 (78.8%)	235 (72.1%)	−6.7%	0.018
**Thiazides (%)**	78 (18.9%)	80 (19.5%)	+0.6%	0.912	19 (22.3%)	10 (11.8%)	−10.5%	0.049	59 (18.1%)	70 (21.4%)	+3.3%	0.215
**Digoxin (%)**	49 (11.9%)	51 (12.4%)	+0.5%	0.883	8 (9.4%)	9 (10.6%)	+1.2%	1.000	41 (12.6%)	42 (12.9%)	+0.3%	1.000
**SGLT2i (%)**	76 (18.5%)	128 (31.1%)	+12.6%	0.340	17 (20.0%)	38 (44.7%)	+24.7%	0.001	59 (18.1%)	117 (35.9%)	+17.8%	0.001

**Table 4:** **Changes in left ventricular remodelling and neurohumoral response in patients with heart failure reduced ejection fraction after specialised nursing care vs. patients receiving conventional follow-up**. Acronyms: LVEF: left ventricular ejection fraction; LVEDD: left ventricular end diastolic diameter; LVESD: left ventricular end systolic diameter; LVEDV: left ventricular end diastolic volume; LVESV: left ventricular end systolic volume; NSHF: nurse specialising in heart failure.

	Total of Patients (*n* = 411)			NSHF (Yes) (*n* = 85)			NSHF (No) (*n* = 326)		
	Basal	Final	*p*	Basal	Final	*p*	Basal	Final	*p*
**LVEF (%)**	30.1 ± 6.0	40.9 ± 14.0	0.001	30.0 ± 5.8	43.9 ± 14.4	0.001	30.1 ± 6.1	40.1 ± 13.8	0.008
**LVEDD (mm)**	62.6 ± 8.3	58.3 ± 8.9	0.001	62.4 ± 7.9	56.5 ± 8.1	0.002	62.6 ± 8.5	58.9 ± 8.1	0.001
**LVESD (mm)**	52.7 ± 9.1	46.1 ± 10.1	0.001	52.8 ± 8.9	40.5 ± 9.7	0.003	52.7 ± 9.2	47.7 ± 9.8	0.001
**LVEDV (mL)**	163.8 ± 60.4	143.2 ± 67.8	0.001	170.1 ± 59.2	153.3 ± 88.9	0.048	161.7 ± 60.8	139.7 ± 58.9	0.001
**LVESV (mL)**	112.1 ± 44.5	87.9 ± 55.6	0.001	115.2 ± 42.8	99.8 ± 72.9	0.049	111.1 ± 45.1	83.4 ± 46.9	0.001
**E/e’**	13.4 ± 5.7	11.4 ± 4.5	0.017	14.1 ± 5.7	13.8 ± 5.6	0.860	13.0 ± 5.7	10.3 ± 3.5	0.006
**PSAP (mmHg)**	30.7 ± 10.0	29.4 ± 10.0	0.260	33.3 ± 10.4	27.6 ± 9.4	0.053	29.9 ± 10.2	29.9 ± 9.8	0.924
**NT-proBNP (pg/mL)**	10,524.2 ± 23,051.1	7108.2 ± 15,127.9	0.214	9596.5 ± 13,056.4	6110.4 ± 17,018.5	0.158	7785.2 ± 8501.9	7406.2 ± 14,544.7	0.666
**CA-125 (U/mL)**	61.8 ± 105.8	30.7 ± 52.2	0.059	31.4 ± 48.8	18.4 ± 24.2	0.238	63.4 ± 111.2	37.4 ± 83.1	0.117

## Data Availability

The data presented in this study are available upon request from the corresponding author.

## Abbreviations

The following abbreviations are used in this manuscript:

HF	Heart failure.
HFrEF	Heart failure and reduced ejection fraction.
HFUs	Heart failure units.
LVEF	Left ventricular ejection fraction.
NSHF	Nurse specialising in HF.
